# A Novel Selective JAK2 Inhibitor Identified Using Pharmacological Interactions

**DOI:** 10.3389/fphar.2018.01379

**Published:** 2018-12-04

**Authors:** Tony Eight Lin, Wei-Chun HuangFu, Min-Wu Chao, Tzu-Ying Sung, Chao-Di Chang, Yi-Ying Chen, Jui-Hua Hsieh, Huang-Ju Tu, Han-Li Huang, Shiow-Lin Pan, Kai-Cheng Hsu

**Affiliations:** ^1^Ph.D. Program for Cancer Molecular Biology and Drug Discovery, College of Medical Science and Technology, Taipei Medical University and Academia Sinica, Taipei, Taiwan; ^2^Graduate Institute of Cancer Molecular Biology and Drug Discovery, College of Medical Science and Technology, Taipei Medical University, Taipei, Taiwan; ^3^Ph.D. Program in Biotechnology Research and Development, Taipei Medical University, Taipei, Taiwan; ^4^Institute of Bioinformatics and Systems Biology, National Chiao Tung University, Hsinchu, Taiwan; ^5^Kelly Government Solutions, Research Triangle Park, NC, United States; ^6^School of Pharmacy, College of Medicine, National Taiwan University, Taipei, Taiwan; ^7^Biomedical Commercialization Center, Taipei Medical University, Taipei, Taiwan

**Keywords:** selective inhibitor, JAK2, virtual screening, docking, pharmacological interaction

## Abstract

The JAK2/STAT signaling pathway mediates cytokine receptor signals that are involved in cell growth, survival and homeostasis. JAK2 is a member of the Janus kinase (JAK) family and aberrant JAK2/STAT is involved with various diseases, making the pathway a therapeutic target. The similarity between the ATP binding site of protein kinases has made development of specific inhibitors difficult. Current JAK2 inhibitors are not selective and produce unwanted side effects. It is thought that increasing selectivity of kinase inhibitors may reduce the side effects seen with current treatment options. Thus, there is a great need for a selective JAK inhibitor. In this study, we identified a JAK2 specific inhibitor. We first identified key pharmacological interactions in the JAK2 binding site by analyzing known JAK2 inhibitors. Then, we performed structure-based virtual screening and filtered compounds based on their pharmacological interactions and identified compound NSC13626 as a potential JAK2 inhibitor. Results of enzymatic assays revealed that against a panel of kinases, compound NSC13626 is a JAK2 inhibitor and has high selectivity toward the JAK2 and JAK3 isozymes. Our cellular assays revealed that compound NSC13626 inhibits colorectal cancer cell (CRC) growth by downregulating phosphorylation of STAT3 and arresting the cell cycle in the S phase. Thus, we believe that compound NSC13626 has potential to be further optimized as a selective JAK2 drug.

## Introduction

The JAKs family consists of four enzymes in mammalian cells: JAK1, JAK2, JAK3, and TYK2 (Menet et al., 2013). These enzymes are part of the JAK/STAT pathway that is activated by cytokines and induce a cascade of signals for development or homeostasis of an organism (Aaronson and Horvath, 2002; Rawlings et al., 2004). The JAK kinases can be thought of as an intermediary between a cytokine signal and the phosphorylated transcriptional factor STAT. A JAK cytokine receptor is composed of many subunits, with some chains associated with a specific JAK isozyme (Banerjee et al., 2017). The carboxy-terminal of JAK contains a JAK homology (JH)1 domain, which is the location of the tyrosine kinase domain (Alicea-Velazquez and Boggon, 2011). This domain is preceded by a pseudokinase domain (JH2) that does not contain key residues for phosphotransfer (Alicea-Velazquez and Boggon, 2011). Instead, this domain is thought to regulate the catalytic activity of the JH1 domain (Alicea-Velazquez and Boggon, 2011).

**GRAPHICAL ABSTRACT:**
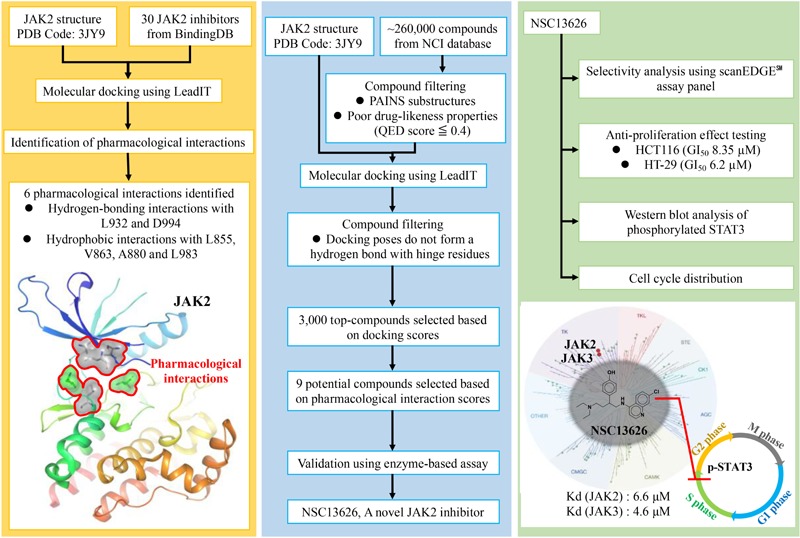
Pharmacological interactions of the JAK2 binding site was established. Next, an *in silico* screening for novel JAK2 inhibitors was performed. Selected compounds were validated using enzyme-based assays. This method identified a novel JAK2 inhibitor.

JAKs are found in virtually all cell types. JAK1 and JAK2 have broad functions, such as hematopoiesis, growth, and neural development, whereas JAK3 and TYK2 primarily regulate the immune response (O’Shea et al., 2013). As a result, aberrant JAK/STAT signaling can lead to various diseases, such as cancer, inflammation and autoimmune diseases (Mascarenhas et al., 2012; O’Shea et al., 2013; Banerjee et al., 2017). JAK1 has been implicated with different kinds of acute leukemia or B-cell lymphoma dependent on mutated sites, JAK2 mutation was usually associated with thrombocytosis, myelofibrosis, leukemia, and lymphoma and increased JAK3 signaling can result in T-cell acute lymphocytic leukemia (O’Shea et al., 2013; Zhang et al., 2018). Additionally, inflammatory cytokines within the tumor microenvironment also play crucial roles in the majority of solid tumors by activating the JAK/STAT3 pathway. Excessive JAK/STAT3 signaling promotes cancer cell proliferation, survival, angiogenesis, tumor metabolism, and antitumor immunity suppression (Buchert et al., 2016; Zhang et al., 2018). Therefore, a concentrated effort has been put in identifying effective JAK inhibitors.

Among the JAK family, JAK2 is an important target for cancer treatment due to its role in cell growth and survival (Verma et al., 2003). The common JAK2 V617 mutation in Philadelphia chromosome-negative myeloproliferative neoplasms (MPNs) spurred the development of JAK2 inhibitors (Baxter et al., 2005; Kralovics et al., 2005). However, a key impediment to developing a specific JAK2 inhibitor is the similarity of the ATP binding site between JAK family members (Mascarenhas et al., 2012; Buchert et al., 2016; Banerjee et al., 2017). Current approved JAK inhibitors include Ruxolitinib, Tofacitinib, Baricitinib, and Oclacitinib which target JAK1/2, JAK1/3, JAK1/2, and JAK1, respectively (Mesa et al., 2012; Wu et al., 2015; Leroy and Constantinescu, 2017). The use of these inhibitors toward the JAK family have brought successful treatment for suffers of myelofibrosis, polycythemia vera, rheumatoid arthritis, pruritus or MPN (Leroy and Constantinescu, 2017). However, Ruxolitinib and Tofacitinib have off-target kinase interactions, which can lead to unwanted side effects (Zhou et al., 2014; Wu et al., 2015). For instance, it has been reported that Ruxolitinib will cause thrombocytopenia, anemia and slight immunosuppression (Leroy and Constantinescu, 2017), while Tofacitinib has shown side effects such as anemia and neutropenia (Wu et al., 2015). Baricitinib has been approved by the European Medical Agency for rheumatoid arthritis; in contrast, the US Food and Drug Administration (FDA) asked for the additional clinical data to clarify safety concerns (Schwartz et al., 2017). It is thought that a specific JAK2 inhibitor can reduce the side effects seen with the current generation of JAK inhibitors (Leroy and Constantinescu, 2017). Therefore, there is still a great demand for a JAK2 specific inhibitor.

Although JAK2 mutations are absent in the majority of solid tumors (Lee et al., 2006; Zhao and Moch, 2008; Herreros-Villanueva et al., 2010), mounting evidences suggest that aberrant JAK2 signaling has an essential role in solid tumors such as colorectal cancer, breast cancer, lung cancer, prostate cancer, etc., (Harry et al., 2012; Zhang et al., 2018). Colorectal cancer is the third leading cause of cancer death worldwide and our poor understanding of its biological molecular mechanisms has yielded little therapeutic results (Miller et al., 2016). Recently, reports have found that inhibition of the JAK2/STAT3 pathway induces cell cycle arrest and apoptosis in colorectal cancer (CRC) cells (Zhang et al., 2018). JAK2 inhibition leads to upregulation of Bax, downregulation of Bcl-2 and loss of mitochondrial membrane potential, which triggers apoptosis in CRC cells (Du et al., 2012). Decreasing the JAK2/STAT3 pathway using the histone deacetylase inhibitor, Trichostatin A, causes CRC cell arrest in the G1 phase, followed by apoptosis (Xiong et al., 2012). These findings indicate that a JAK2 inhibitor may be a beneficial target for the treatment of CRC.

In this study, we used a structure-based virtual screening approach to identify new JAK2 inhibitors with novel scaffolds. In general, this approach docks compounds into a structure of the target protein and a selection of potential inhibitors is made based on the docking scores (Meng et al., 2011). While there has been successes with virtual drug screening, high accuracy binding affinity is still far from certain (Cheng et al., 2012), resulting in a low hit rate. Identifying interactions essential for ligand-target binding is critical and offers a way to improve the structure-based virtual screening hit rate (Cheng et al., 2012). For example, most kinase inhibitors consistently form hydrogen bonds with hinge residues (McGregor, 2007), which can be regarded as pharmacological interactions. Thus, filtering compounds based on their docking scores and pharmacological interactions provides a useful strategy to better understand the binding mechanism and to improve the hit rate for potential kinase inhibitors. Herein, we identified JAK2 pharmacological interactions using known JAK2 inhibitors. Compounds from the National Cancer Institute (NCI) database were then docked and filtered based on the binding scores and interactions with key residues. Finally, the top virtual hits were selected for enzymatic and cellular studies. Our efforts identified a promising lead JAK2 inhibitor.

## Materials and Methods

### Chemical and Reagents

Compounds, such as NSC13626 were requested from National Institute of Health (Bethesda, MA, United States). HCT116 CRC line was obtained from Bioresource Collection and Research Center (Hsinchu, Taiwan). McCoy’s 5A cell culture medium, sulforhodamine B (SRB), dimethyl sulfoxide, ethylenediaminetetraacetate, propidium iodide, and Triton X-100 purchased from Sigma (St. Louis, MO, United States). Trichloroacetic acid (TCA), acetic acid, Trizma base, Tris–HCl, sodium chloride, sodium dodecyl sulfate, ethanol, citric acid, and Na_2_HPO_4_ were purchased from Wako Pure Chemical Industries (Osaka, Japan). Cocktail protease inhibitor was obtained from Calbiochem Research Biochemicals (Merck, Burlington, MA, United States). RNase A was purchased from Bioshop (Ontario, Canada).

### Molecular Docking

The docking software Sybyl (Certara, 2017), CDOCKER (BIOVIA Discovery Studio, 2016a), iGEMDOCK (Hsu et al., 2011), and LeadIT (LeadIT, 2011) were used to determine the most appropriate program for this study. LeadIT is based on an interaction model called LUDI (Bohm, 1994), which calculates interaction sites within the protein’s binding site, fits fragments into the interaction site and proposes an alignment for the docked fragments. LeadIT produces a match score for each interaction between a docking pose of a compound and protein residues to represent relative strength of each interaction. Finally, a total score is produced for each compound. All docking parameters used default settings.

Compounds were docked using the most effective molecular docking program for this study, LeadIT (LeadIT, 2011). In addition, LeadIT contains a user-friendly interface and guidelines to prepare molecular docking (LeadIT, 2011). The co-crystal structure of JAK2 (PDB ID: 3JY9) was obtained from the RCSB Protein Data Bank (PDB; Berman et al., 2000). The structure was selected based on the following criteria: (1) a crystal structure with a resolution lower than 2.5 Å, (2) a crystal structure containing a potent co-crystal ligand (*K*_i_: 1 nM) (Wang et al., 2009), and (3) a crystal structure with no known mutation. The binding site was prepared using the location of the co-crystallized ligand. A docking radius of 10 Å was selected from the co-crystalized ligand to avoid missing potential inhibitors with different interactions. Water atoms in the binding site were removed. All docked compounds were protonated in aqueous solution. Docking was performed using the FlexX docking module of LeadIT. The docking strategy used the hybrid (enthalpy and entropy) approach. The default settings were used for the scoring parameters.

### Pharmacological Interactions

A set of diverse JAK2 inhibitors were collected to identify pharmacological interactions. Known JAK2 inhibitors were obtained from BindingDB (Liu et al., 2007) and inhibitors with IC_50_ values > 1 μM were removed. The “Diverse Molecules” component from Pipeline Pilot (BIOVIA Discovery Studio, 2016b), which selects diverse compounds based on the maximum dissimilarity method, was used to select diverse JAK2 inhibitors. This module provides an unbiased selection of structures. The selected inhibitors were docked to the JAK2 binding site using the methods mentioned above. Interactions between the compound and binding site residues were obtained using the “analyze non-bond interactions” component in pipeline pilot (BIOVIA Discovery Studio, 2016b). Three types of interactions were analyzed. Hydrogen bonds had a maximum distance of 3.4 Å and 3.8 Å for strong and weak bonds, respectively. The hydrophobic interactions in this study includes Pi-Pi, alkyl, Pi-Alkyl, and Pi-Sigma interactions. The maximum distance for each type is 6 Å, 5.5 Å, 5.5 Å, and 4 Å, respectively. The charge-charge interactions are formed between atoms with opposite whole or fractional formal charges that are within the maximum distance cutoff (5.6 Å). Pharmacological interactions were developed by analyzing the non-bond interactions from known JAK2 inhibitors. An interaction was defined as a pharmacological interaction if ≥50% of the known JAK2 inhibitors formed an interaction with a residue (Yang and Shen, 2005).

### Selection of Potential Inhibitors

Compounds from the NCI database were selected for screening. The database contains roughly 260,000 compounds. Compounds that are potential pain assay interference compounds (PAINS) were filtered (Baell and Holloway, 2010). Compounds with PAINS substructures often cause false-positive assay. The PAINS substructures were obtained from ZBH Center for Bioinformatics Hamburg. Compounds showing poor drug-likeness properties were removed. The “Drug Likeness” component from Pipeline Pilot was used to estimate drug-likeness properties and calculate Quantitative Estimate of Drug-Likeness (QED) scores for compounds. Compounds with ≦0.4 QED scores were filtered. The QED score is a metric that assess drug-likeness and can: (1) rank a large number of compounds, even if some fails the Lipinski Rule of Five, (2) identify cases where a generally unfavorable property can be tolerated, (3) give a more realistic description of a compound quality (Bickerton et al., 2012). Compounds that do not contain a cyclic ring with a nitrogen were also removed because most kinase inhibitors use the functional group to form hydrogen bonds with hinge residues (McGregor, 2007). 3,000 top compounds ranked based on FlexX scores were selected for pharmacological analysis. The compounds were re-ranked according to their pharmacological interaction scores. The pharmacological interaction score is calculated as follows: for a compound *i*, its pharmacological interaction score, *S*(*i*), is defined as

$$S(\underline{i})\;=\;N(i)+{\left(-0.01\right)}^{*}D(i)$$

where *N*(*i*) is the number of pharmacological interactions the compound forms and *D*(*i*) is the docking score of compound *i* generated using FlexX. The coefficient of -0.01 is used to normalize the docking score from 0 to 1. Finally, potential JAK2 inhibitors were selected for further testing based on their pharmacological ranking and compound availabilities.

### Molecular Field Map

The molecular force fields were used to examine important regions essential to compound activity. The fields were developed by the computational program Forge (Cresset, 2015). The Forge software was used to analyze structure-activity relationship (SAR) because of the following advantages: (1) it can produce a 3D map with electrostatic field and surface properties between a ligand and protein and (2) it can generate force fields that show activity cliffs, which represent compound pairs where structural differences can cause changes in activity. An activity cliff was developed and summarized for compound NSC13626 and its analogs to produce a global activity atlas model. All molecular force fields were generated using the default settings.

### Compound Similarity Matrix

The structures of inhibitor NSC13626 and diverse JAK2 inhibitors were compared by generating an atom-pair fingerprint for each compound using the RDKit Fingerprint tool in KNIME (Berthold et al., 2007). The similarity matrix and hierarchal clustering was created using the program Morpheus.^1^ Compounds were sorted based on similarity and their Pearson correlation coefficient. Compounds with high or low similarity are colored red and blue, respectively.

### *K*_d_ Measurement

*K*_d_ values were determined by DiscoverX using their bead-based competition assay KinomeScan^TM^.^2^ The binding affinity (*K*_d_) is a metric for evaluating inhibitor selectivity. This is a fast and reliable screening assay for compounds against a customizable panel of kinases. In short, kinases are expressed on phage and immobilized by beads via active site directed ligands. The assay tests if a compound can disrupt a high affinity ATP-mimic probe and the kinase of interest. Selected compound binding affinity is tested by premixing with compounds with kinases and assayed for kinase binding to immobilized ligands. A standard dose-response curve using the Hill equation is used to calculate binding constraints (Sonoshita et al., 2018). Compounds were then assessed using the DiscoverX *scan*Edge assay. This assesses compound selectivity against a panel of kinases distributed throughout the kinome. Two replicates were performed and averaged to obtain *K*_d_ measurement.

### Kinase Inhibition Measurement

The biochemical kinase assay was performed by ThermoFisher Scientific using their Z’LYTE kinase activity assay.^3^ The test compounds were screened in 1% DMSO (final) and combined with a kinase mixture diluted to 2X working concentration with a kinase buffer. The development reagent solution and ATP solution were diluted to appropriate concentration and all reagents are mixed and incubated for 1 h. Finally, the reactions are measured on a fluorescence plate reader. The result of the kinase inhibition assay is an average of two replicates.

### Cell Proliferation Assay (SRB Assay)

The anti-proliferative potency of compound NSC13626 was evaluated using the SRB assay (Vichai and Kirtikara, 2006). CRC lines, HCT116 and HT-29, were both cultured in McCoy’s 5A medium and seeded in 96-well plates (5,000 cells/well) overnight and fixed with 10% TCA representing the initial cell density. After treatment with different concentrations of vehicle (0.1% DMSO, dimethyl sulfoxide) or JAK2 inhibitor, NSC13626, for 48 h, cells were fixed with TCA and. stained with 0.4% SRB (Sigma, St. Louis, MO, United States) in 1% acetic acid, and then washed by 1% acetic acid three times. Cell population (dye-containing cells) was lysed by 10 mM Trizma base. Then the absorbance was read at 515 nm. Non-linear regression analysis was used to determine concentrations that caused a 50% reduction in cell growth (GI_50_, 50% of growth inhibition).

### Western Blot Analysis

HCT116 cells were treated with the indicated concentrations for 48 h, and then were harvested and lysed with RIPA buffer (50 mM Tris–HCl, pH 7.4, 150 mM sodium chloride, 1 mM ethylenediaminetetraacetic acid (EDTA), 1% NP-40, 1% sodium deoxycholic acid, 0.1% sodium dodecylsulfate (SDS), 1 mM phenylmethylsulfonyl fluoride and protease inhibitor cocktails) for 30 min. Cell lysates were centrifuged at 13,000 rpm at 4°C for 30 min. Protein was quantified by BCA Protein Assay Kit (Thermo scientific, Waltham, MA, United States). The equal protein (50 μg) was loaded and separated by electrophoresis and then transferred to polyvinylidene difluoride (PMSF) membrane for 2 h. The membranes were slowly shaken in 5% non-fat milk with PBS for 1 h at room temperature. After that, the membranes were incubated with the indicated primary antibodies at 4°C overnight and the secondary antibodies (Santa Cruz Biotechnology, Dallas, TX, United States) were applied for 1 h at room temperature. Finally, the signals were detected with the enhanced chemiluminescence. p-STAT3, t-STAT3, p-AKT, t-AKT, p-ERK, t-ERK antibodies were purchased form Cell Signaling Technologies (Danvers, MA, United States) and Actin antibody was from Merck Millipore (Burlington, MA, United States).

### Cell Cycle Analysis

HCT116 cells were seeded in 6-well plates (30,000 cells/well) and treated with 0.1% DMSO (vehicle) or 1, 10 μM NSC13626 for 48 h. After trypsinized and fixed in ice-cold 75% (v/v) ethanol at -20°C overnight. Cells were washed with PBS (phosphate-buffered saline), resuspended in DNA extraction buffer (0.2 M Na_2_HPO_4_, 0.1 M citric acid; pH 7.8) for 30 min. Then cells were centrifuged, removed the supernatant, and then stained with propidium iodide solution (PI, 80 μg/ml; 0.1% Triton X-100; 100 μg/m RNase A in PBS). DNA content was analyzed with BD Accuri and C6 Software (BD Biosciences).

### Statistical Analysis

*In vitro* experiments were acquired at three independent times. SRB results are presented as means with standard deviation (SD). Statistical analysis was conducted by Prism 7.0 software, and the significance between two groups was determined by Student’s *t*-test. One asterisk indicates *P* < 0.05.

## Results

### Identification of Pharmacological Interactions

In this study, we performed a structure-based virtual screening to identify new JAK2 inhibitors. The JAK2 kinase structure contains two lobes, a β-sheet N-lobe and a C-lobe, that is connected by a hinge (Alicea-Velazquez and Boggon, 2011) (Supplementary Figure 1). The N-lobe is flexible and facilitates activation and regulation of ATP/ADP binding and release. The JAK2 binding site contains several important residues, such as hinge residues Glu930, Leu932, and A-loop residues Asp994 and Phe995 (Hitoshi et al., 2010) (Supplementary Figure 1A). The volume of the binding site was calculated using CASTp to be 756 Å^3^ (Tian et al., 2018) (Supplementary Figure 1B). Understanding the ligand-protein interactions is the key to identifying an effective hit (Cheng et al., 2012). To achieve this, we first looked to identify pharmacological interactions between the binding site residues and JAK2 inhibitors. We selected known active JAK2 inhibitors obtained from BindingDB (Liu et al., 2007). Inhibitors with an IC_50_ value of 1 μM or less were selected. The “Diverse Molecules” component from Pipeline Pilot was used to select JAK2 inhibitors with diverse structures. This module provides an unbiased selection of structures. Finally, 30 inhibitors with diverse structures were selected for the interaction analysis.

To assess the performance of the docking software to be used in this study, we mixed the 30 selected inhibitors with 990 randomly selected compounds from the Available Chemical Directory (ACD; Bissantz et al., 2000) and docked this set into JAK2 using four different softwares: LeadIT (LeadIT, 2011), CDOCKER (BIOVIA Discovery Studio, 2016a), iGEMDOCK (Hsu et al., 2011), and SYBYL (Certara, 2017). The method with the best true positive hit rate will be selected. We identified the true positive hit rate as *I*/*T* (%), where *I* is the number of the JAK2 inhibitors among the *T* highest-ranking compounds. When we ranked the docking scores of the JAK2 inhibitors, we found that LeadIT produces a better hit rate compared to the other software used (Supplementary Figure 2). To further validate our docking procedure, we performed a redocking experiment of the co-crystal ligand of JAK2 (PDB ID: 3JY9). The docking result was similar to the co-crystal structure (Supplementary Figure 3). This suggest that the docking procedure is effective. Therefore, LeadIT was selected for our docking experiments.

**FIGURE 1:**
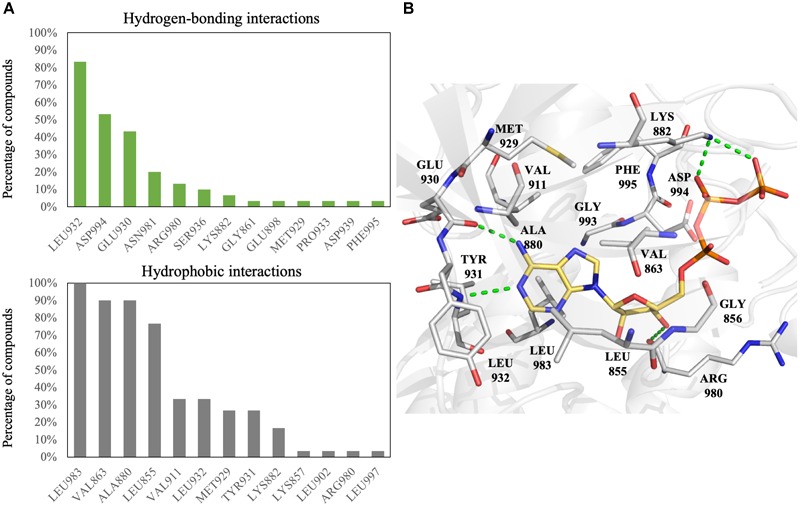
Interactions analysis of JAK2 inhibitors. **(A)** Interactions of JAK2 binding site residues identified from known JAK2 inhibitors. Hydrogen bond (green) and hydrophobic interactions (gray) are graphed as shown. **(B)** Binding pose of ATP (yellow) in JAK2 (gray) and rendered in Pymol. Green dashed lines denote hydrogen bonds. Residues are labeled as shown.

To increase the virtual screening hit rate, we docked the 30 inhibitors into the binding site to analyze their pharmacological interactions. An interaction with a frequency of ≥50 % was considered a key pharmacological interaction (Yang and Shen, 2005). Filtering compounds based on pharmacological interactions can be used to increase our virtual screening hit rate. In total, we found six pharmacological interactions in the JAK2 binding site. The pharmacological interactions include two hydrogen-bonding and four hydrophobic interactions (Figure 1). Roughly 90% of known JAK2 inhibitors form a hydrogen bond with hinge residue Leu932, followed closely by the DFG motif residue Asp994 (Figure 1A). Hydrogen bonds with hinge residues are considered critical for kinase inhibition (McGregor, 2007). The adenosine ring of ATP, when docked into the JAK2 crystal structure, creates hydrogen bonds between the aforementioned hinge residue and residue Glu930 with the heterocyclic nitrogen and the primary amine, respectively (Figure 1B). In addition, known JAK2 inhibitors create at least one hydrogen bond with hinge residues (Supplementary Figure 4). While many inhibitors formed hydrogen bond with Glu930, it did not meet the 50% threshold to be considered a key pharmacological interaction. A hydrogen bond with residue Asp994 is common among JAK2 inhibitors. However, when ATP is docked into the JAK2 binding site, we did not observe a hydrogen bond with residue Asp994. Instead, the phosphate of ATP form hydrogen bonds with residue Lys882 (Figure 1B). We also identified four key hydrophobic interactions: residues Leu983, Ala880, Val863, and Leu855 (Figure 1A). These residues are located near the adenine and ribose structure of ATP and form a hydrophobic pocket, which can be exploited by various heterocyclic structures to stabilize the compounds within the binding site (Zhang et al., 2009). Notably, many FDA approved kinase inhibitors consist of a nitrogen based heterocyclic compound that occupies the hydrophobic pocket as well as forming hydrogen bonds with the hinge. For example, the hinge binding motif for JAK2 inhibitor 5194003 includes a diazaindole structure (Supplementary Figure 4).

To evaluate the accuracy of our positive hits, the inhibitors were then ranked by two methods: docking score and pharmacological interaction score (Supplementary Figure 5). The inhibitor rankings between the two methods varied greatly. Together, this data suggests that ranking compounds based on their pharmacological interactions can identify a potent JAK2 inhibitor.

### Selection and Validation of Potential Inhibitors

After the identification of pharmacological interactions, we virtually screened the NCI database (roughly 260,000 compounds) using the computational docking software LeadIT (LeadIT, 2011). Potential PAINS and compounds with QED score of < = 0.4 were removed. When compared to the Lipinski Rule of Five, the QED score can identify compounds with better drug-like properties; thus false positives may be reduced (Yusof and Segall, 2013).

Furthermore, compounds that do not contain a heterocyclic ring system were filtered because such functional groups typically form hydrogen-bond interactions with hinge residues (Zhang et al., 2009). The remaining compounds were then ranked based on their docking scores. The top ranked compounds (3,000) were selected. Next, these compounds were ranked according to their number of pharmacological interactions with JAK2. Finally, available compounds were requested for *in vitro* testing. In total, our screening yielded nine potential inhibitors (Supplementary Figure 6). The nine potential inhibitors were tested against JAK2 using the ThermoFisher biochemical kinase assay. The kinase profiling was done at 10 μM for the selected compounds. Of the virtually hit compounds, compound NSC13626 showed effective JAK2 inhibition. Compound NSC51563 had an inhibition at 46.1% and did not meet the 50% threshold in our study (Supplementary Figure 6). Because NSC13626 had the best inhibition activity, it was selected for further study.

**FIGURE 2:**
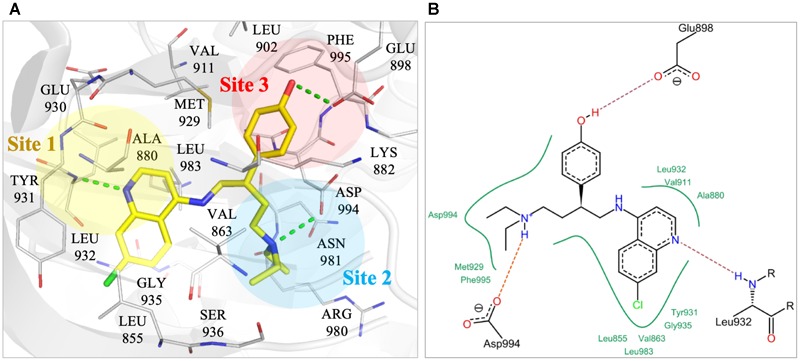
Interactions of compound NSC13626 in JAK2 binding site. **(A)** The docking pose of compound NSC13626 (yellow) in JAK2 (gray). Dashed green line denotes hydrogen bond. Site 1 (yellow), Site 2 (blue), and Site 3 (red) are colored as shown. The docking pose was rendered using Pymol. **(B)** The 2D representation of compound NSC13626 docked in JAK2 shows both hydrogen and hydrophobic interactions. Red dashed line denotes hydrogen bond, green line denotes hydrophobic interactions. 2D representation was created in LeadIT.

To better understand the binding mechanism, we performed an interaction analysis of compound NSC13626 in the JAK2 (PDB ID: 3JY9) binding site (Figure 2). LeadIT produces a match score that represents the relative strength of each interaction. The interactions can be separated into three distinct Sites. Each Site contains a hydrogen bond (Figure 2A). Site 1 contains the 7-chloroquinolin-4-amine moiety, and the cyclic nitrogen creates a hydrogen bond with the nitrogen of the hinge residue Leu932. The match score of this interaction is -4.7. Like ATP, this structure is also stabilized by hydrophobic interactions formed by residues Tyr931, Leu932, and Leu983 (Figure 2B). Site 2 contained the DFG-loop (residues Asp994, Phe995 and Gly996) of JAK2. The butyldiethylamine moiety, which contains a tertiary amine, form a hydrogen bond with the oxygen of the DFG residue Asp994. The hydrogen bond with Asp994 is scored at -8.3, which is stronger than that found with Leu932. The two moieties are connected by a phenol moiety, which occupies Site 3. The hydroxyl group of the phenol moiety is the hydrogen bond donor to the carboxyl group of residue Glu898. This hydrogen bond is scored at -3.7. Site 3 also favors a cyclic compound, forming hydrophobic interactions with the phenol ring. These interactions elucidate compound NSC13626 interactions within the JAK2 binding site.

**FIGURE 3:**
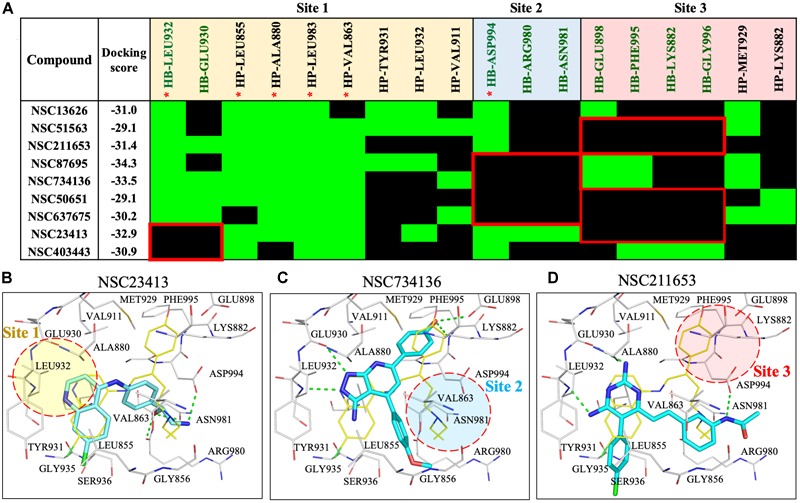
Interaction analysis of virtual screening hits. **(A)** Interaction analysis of virtual screening hits. The docking score for each compound was calculated in LeadIT. Hydrogen-bonding (HB) and hydrophobic (HP) residue interactions are labeled as shown. Residues with asterisk are identified as pharmacological interactions. Common residues with no interactions with inactive compounds are highlighted in red. Docking poses of **(B)** NSC23413, **(C)** NSC734136, and **(D)** NSC211653 in JAK2 (Gray) and rendered in Pymol. Dotted green line denotes hydrogen bond. Dotted circle shows Site with no hydrogen bond. Site is listed as shown.

To understand the increased potency of compound NSC13626 compared to the other screening hits, we compared their interactions (Figure 3). A hydrogen bond at each site appears crucial for effective JAK2 inhibition. Compound NSC13626 created a hydrogen bond with each site. Hydrogen bond formation with hinge residues (Site 1) is deemed crucial for kinase inhibition. Therefore, NSC23413 and NSC403443, while processing a favorable docking score and high pharmacological score in this study, are not effective JAK2 inhibitors (Figures 3A,B). Many of the compounds show different interactions at Site 2 and Site 3. A hydrogen bond with the DFG motif at Site 2 shows positive potency for many JAK2 inhibitors (Figure 1A). The potential inhibitors in this study that lacked this interaction showed weak JAK2 inhibition (Supplementary Figure 7). For example, NSC734136 create hydrogen bonds at Site 1 and Site 3, but not with residue Asp994 at Site 2 (Figure 3C). Meanwhile, compound NSC211653 create hydrogen bonds with Site 1 and Site 2, but not with residue Glu898 at Site 3 (Figure 3D). The interactions at Site 3 show new important interactions when compared to our pharmacological interactions. The potent compound NSC13626 was the only identified hit that created a hydrogen bond with key pharmacological residues Leu932 and Asp994 as well as a hydrogen bond with residue Glu898 at Site 3.

To understand the importance of geometric and spatial locations of our hit compound, we compared NSC13626 to inactive compounds in the JAK2 binding site. The inactive compounds in JAK2 show different interactions with respect to NSC13626. Missing interactions at either site 2 or site 3 greatly reduce their ability for JAK2 inhibition (Supplementary Figures 7A,B). The co-crystal ligand, JZH (PDB ID: 3JY9) is an equipotent inhibitor producing a *K*_i_ of 0.0010 and 0.0055 μM for JAK2 and JAK3, respectively (Wang et al., 2009). The co-crystal ligand JZH occupies all three sites. In addition, ligand JZH contains a phenol that has accesses to Site 3 (Supplementary Figure 7C). This suggests that interactions at all three sites are crucial for identifying potent inhibitors during the drug screening process.

### Selectivity of NSC13626

**FIGURE 4:**
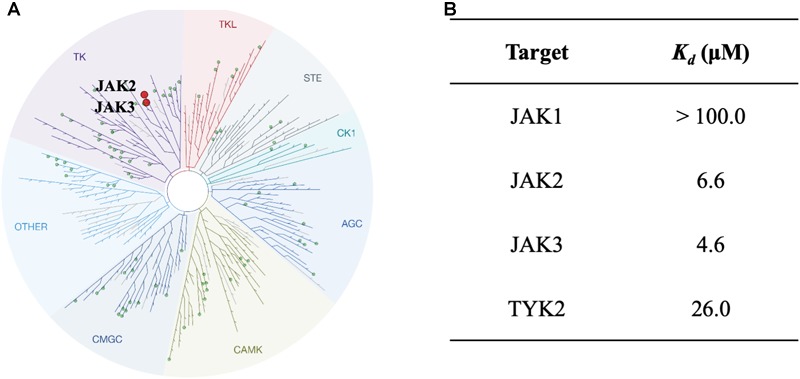
Inhibitor NSC13626 profile data shows JAK2/3 selectivity. **(A)** Results of kinase screening. Inhibitor NSC13626 is specific toward the JAK isozyme when screened against a panel of 97 kinases. Red circle indicates kinase target of NSC13626. The figure was generated using TREEspot (http://treespot.discoverx.com/) **(B)** The *K*_d_ values of NSC13626 were calculated against the JAK family. Inhibitor NSC13626 has the strongest binding affinity toward JAK2 and JAK3 when compared to the JAK isozymes.

To study the selectivity of inhibitor NSC13626, we performed kinase profiling of compound NSC13626 against a panel of 97 different kinases dispersed across the kinome. Using the scanEDGE^SM^ kinase assay panel revealed that among the 97 kinases tested, compound NSC13626 inhibited the JAK isozyme family (Figure 4A). The JAK kinase family consists of four isozymes: JAK1, JAK2, JAK3, and TYK2. Of the four isozymes, NSC13626 has high binding affinity toward JAK2 and JAK3 at 6.6 μM and 4.6 μM, respectively. In contrast, JAK1 and TYK2 showed reduced binding affinity to NSC13626 (Figure 4B).

### Structure-Activity Relationship Analysis of NSC13626

**FIGURE 5:**
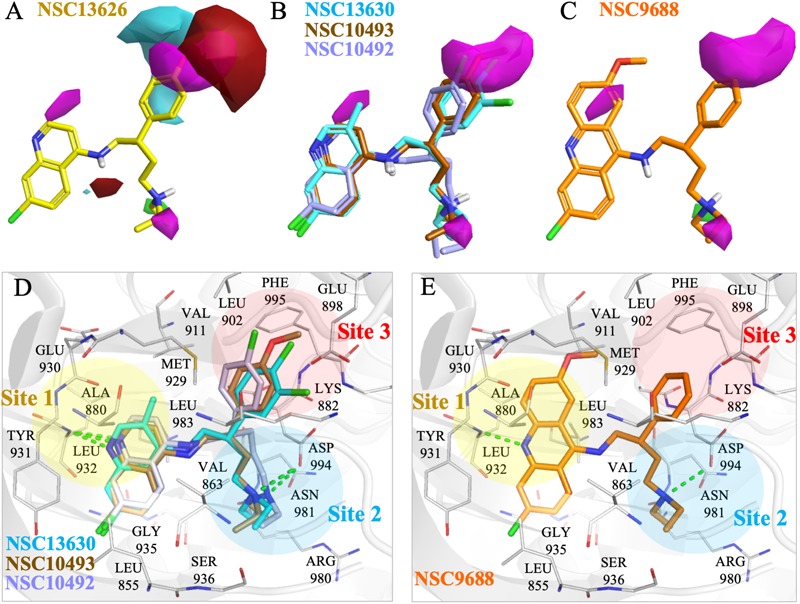
Molecular force field analysis of NSC13626 and its analogs in JAK2 binding site. The structure activity relationship between compound **(A)** NSC13626 and **(B,C)** its analog shows different interactions in the JAK2 binding site using the Forge software. Areas of interest are colored as: red, more positive electrostatics increases activity, blue, more negative electrostatics increases activity, and pink, steric bulk in this position reduces activity. **(D,E)** The binding poses of analogs in JAK2 (gray) rendered in Pymol. Hydrogen bonds are denoted as dashed green line. Sites and residues are labeled as shown.

To elucidate the SAR of NSC13626, we obtained NSC13626 analogs from the NCI database (Supplementary Figure 8). Enzymatic assays did not show effective inhibitory activities for the eight analogs selected. A molecular field of the JAK2 binding site was developed using the software Forge (Cresset, 2015) to identify structural differences that can affect inhibitory interactions. The map produced is an “activity cliff summary,” which details regions where more positive or negative electrostatics and steric clashes can occur to reduce activity (Figure 5). The analogs were found to occupy similar interactions at sites 1 and 2. However, interactions at Site 3 vary between NSC13626 and its analogs, which suggests its importance in selectivity. The hydroxyl group of NSC13626 form a hydrogen bond to the carboxyl group of residue Glu898, which is located within a region that favors negative electrostatic potential (Figure 5A). In contrast, the analogs do not favorably occupy Site 3 (Figures 5B,C). Analogs that contain a chloride or methoxy moiety are sterically hindered. In addition, they are unable to form a hydrogen bond with Glu898 (Figures 5B,D). Meanwhile, the analog NSC9688 contains a benzyl group, which subsequently cannot extend into Site 3 to form a hydrogen bond with Glu898 (Figures 5C,E). Furthermore, NSC9688 contains three cyclic rings at Site 1, unlike NSC13626, which contains two. This is much larger and unfavorable in Site 1 (Figure 5E).

**FIGURE 6:**
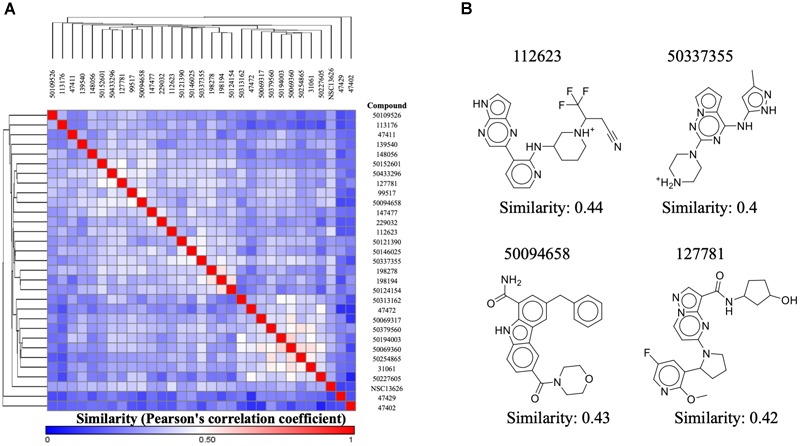
Inhibitor NSC13626 is a novel structure. **(A)** A heatmap of structure similarity between inhibitor NSC13626 and known JAK2 inhibitors from BindingDB. Most similar or least similar compounds are colored red and blue, respectively. **(B)** Most similar compounds and their 2D structures are listed as shown.

Our aim in this study was to identify a novel JAK2 inhibitor. As a result, we compared the structure of inhibitor NSC13626 with known JAK2 inhibitors obtained from BindingDB (Figure 6A). We focused three set, each with a scaffold core with modifications (Ledeboer et al., 2009; Lim et al., 2011; Simov et al., 2016). These three sets contain the most JAK2 inhibitors from BindingDB. A hierarchal clustering approach was used for the compound structures and a Pearson’s correlation measured the similarity between the rows/columns and is represented as a heatmap. In short, we collected 128 known JAK2 compound structures as well as compound NSC13626. The average intra-class similarity, which describes compounds that in the group with similar structures, was calculated to be 0.6. In contrast, the average inter-class similarity, which details compounds with dissimilar structures, was calculated to be 0.4. Therefore, compounds with 0.6 similarity score or higher would have similar scaffolds, while compounds exhibiting novel scaffolds with a score of 0.4 or lower. Compound NSC13626 was rated no higher than 0.44 when compared to the known JAK2 inhibitor structures, suggesting that NSC13626 is a novel scaffold.

To determine if compound NSC13626 has a novel scaffold compared to known JAK2 inhibitors, we calculated a Pearson correlation coefficient to obtain compound similarity scores. We used three sets, each focused on a scaffold core with modifications These three sets contain the most JAK2 inhibitors from BindingDB. In short, we collected 128 known JAK2 compound structures as well as compound NSC13626. The average intra-class similarity, which describes compounds that in the group with similar structures, was calculated to be 0.6. In contrast, the average inter-class similarity, which details compounds with dissimilar structures, was calculated to be 0.4. Therefore, compounds with 0.6 similarity score or higher would have similar scaffolds, while compounds exhibiting novel scaffolds with a score of 0.4 or lower (Figure 6B). Compound NSC13626 was rated no higher than 0.44 when compared to the known JAK2 inhibitor structures, suggesting that NSC13626 is a novel scaffold.

### *In vitro* Studies of Inhibitor NSC13626

**FIGURE 7:**
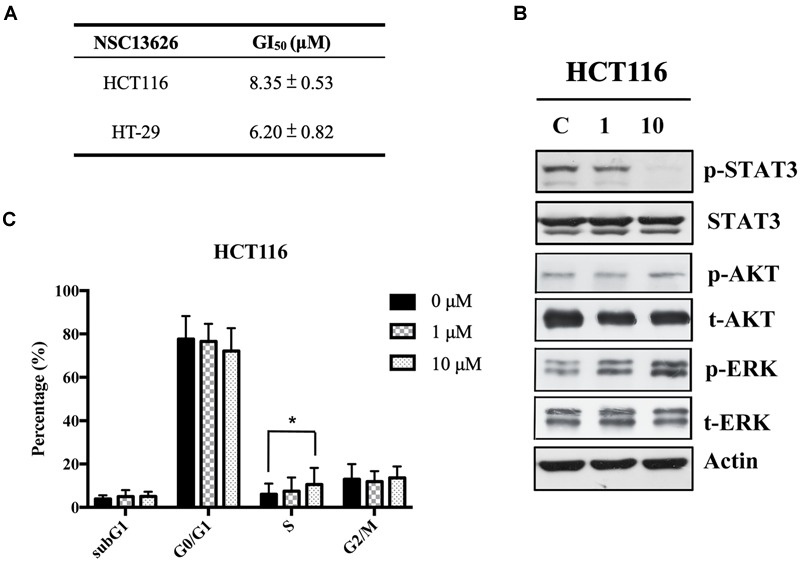
Inhibitor NSC13626 displays favorable *in vitro* efficacy. **(A)** Inhibitor NSC13626 has favorable GI_50_ in HCT116 and HT-29 colorectal cancer cells. **(B)** Western blot analysis of phosphorylated STAT3, a JAK2 downstream signaling protein, p-AKT, p-ERK, total STAT3, total AKT, and total ERK with actin as an internal control. HCT116 cells were treated with indicated treatments. The basal condition was in the absence of FBS (fetal bovine serum), while vehicle and treatment groups were incubated with 0.1% DMSO (as vehicle), 1 μM and 10 μM NSC13626, respectively, with 10% FBS for 48 h. **(C)** HCT116 cells were treated with vehicle, 1 μM and 10 μM NSC13626 for 48 h. The cell cycle distribution was determined by propidium iodide staining. Data was conducted at least three independent experiments and statistical analysis was estimated by *t*-test. [^∗^] indicates *p* < 0.05. The figure was generated using Prism.

The anti-proliferation effect of inhibitor NSC13626 was tested *in vitro* using human CRC. The GI_50_ (50% growth inhibition) were determined for inhibitor NSC13626 in HCT116 (8.35 ± 0.53 μM) and HT-29 (6.20 μM ± 0.82 μM) cell lines (Figure 7A). Since persistent activation of STAT3 is oncogenic and is prevalent in a wide variety of human cancers, such as CRC (Xiong et al., 2012), we focused on the JAK2/STAT3 pathway. STAT3, the main downstream target of JAK2, plays an essential role in proliferation and survival in colon cancer-initiating cells (Lin et al., 2011). Additionally, reducing the JAK2/STAT3 pathway can induce colorectal cell apoptosis (Du et al., 2012), while overexpression of IL-6 can induce the JAK2/STAT3 signaling pathway to enhance progression of CRC (Zhang et al., 2018). To confirm the target of NSC13626 in cellular conditions, we evaluated the levels of p-STAT3. The protein analysis results showed that inhibitor NSC13626 was able to dramatically reduce the levels of STAT3 phosphorylation in a dose-dependent manner at 48 h, certifying its potent enzymatic activity (Figure 7B). The activation of the JAK/STAT signaling pathway may activate other downstream signaling pathways, such as PI3K/AKT and the RAS/ERK (Vainchenker and Constantinescu, 2013). Nonetheless, the levels of p-AKT and p-ERK showed no changes with NSC13626 treatment, suggesting that NSC13626 was specific to JAK2/STAT3 signaling in HCT116 cells (Figure 7B). Cell growth can also be disrupted with STAT3 inhibition (Turkson et al., 2005). We analyzed the cell cycle distribution of HCT116 cells treated with inhibitor NSC13626 at 1 μM and 10 μM for 48 h. A majority of the cells were arrested in the S phase (*P* < 0.05) when compared to the vehicle group (Figure 7C). These observations demonstrated the anti-tumor activity of inhibitor NSC13626 and suggests it can be used as a therapeutic against CRC. Further studies will need to be performed to modify potency and selectivity toward JAK2.

## Discussion

In this study, we performed a structure-based virtual screening of the NCI database to identify novel and specific JAK2 inhibitors. While *in silico* screening for JAK1 or JAK2 inhibitors using the NCI database have been carried out previously (Kiss et al., 2009, 2016), we devised a new strategy to increase the hit rates for potential inhibitors. First, to increase our hit rate, we identified pharmacological interactions by docking known JAK2 inhibitors. Next, the compounds of the NCI database were docked and filtered based on their docking scores and pharmacological interactions to identify inhibitors with a novel scaffold. Our methods yielded one compound, NSC13626, that contained a novel structure compared to known JAK2 inhibitors and showed high efficacy in enzymatic and cellular assays studies.

We first elucidated pharmacological interactions of the JAK2 binding site. We identified six key residues. Two (Leu932 and Asp994) were important for hydrogen bonds, while four (Leu855, Val863, Ala880, and Leu983) were used for hydrophobic interactions (Figure 1). Residue Leu932 was especially important, since hinge residue interactions are necessary in many types of kinase inhibitors (McGregor, 2007) and hydrophobic interactions are exploited by heterocyclic structures to stabilize the compounds within the binding site (Zhang et al., 2009). To increase the hit rate, the pharmacological interactions were used as an extra screening criterion. Our method was validated by combining 30 known JAK2 inhibitors with 990 randomly selected compounds from the ACD (Bissantz et al., 2000). Ranking the compounds based on the pharmacological interaction increased the scored rank of the 30 known JAK2 inhibitors (Supplementary Figure 5).

The JAK2 binding site was separated into three sites based on the interactions with NSC13626. Importantly, each site contains a hydrogen bond that appears to be key in inhibiting JAK2 in this study (Figure 3). The hinge residue Leu932 (Site 1) and Asp944 (Site 2), were identified as key residues (Figure 1). ATP did not form a hydrogen bond with residue Asp994 (Figure 1B). This suggests that a different area within the binding pocket that potential inhibitors may exploit. Site 3 proved just as important for JAK2 inhibition, with residue Glu898 an important residue for NSC13626. Indeed, the virtual hits that lacked interactions with Site 3 did not show effective JAK2 inhibition (Figure 3). Furthermore, Site 3 does not favor a large moiety (Figure 5). The phenol moiety of NSC13626 creates a hydrogen bond with Glu898. A smaller substituent and a polar moiety that occupies this hydrophobic region may offer a way to increase JAK2 selectivity.

It is essential for JAK2 inhibitors to not only show potent enzymatic inhibition, but selectivity toward JAK2 to prevent off target side effects, such as peripheral neuropathy, anemia, and thrombocytopenia (Zhao et al., 2016). This is a difficult task due to the conserved ATP site for the JAK family (Alicea-Velazquez and Boggon, 2011). The FDA-approved kinase inhibitor ruxolitinib targets JAK1 and JAK2, while tofacitinib can selectively target JAK1-3 (Mesa et al., 2012; Wu et al., 2015). We found that the identified inhibitor, NSC13626, has high selectivity toward JAK2 and JAK3 and may circumvent the off-target concerns seen with current JAK2 inhibitors (Figure 4).

To analyze selectivity, we docked NSC13636 to JAK isozymes and compared the docking poses (Supplementary Figure 9A). The docking results show that the docking pose of NSC13626 in JAK3 is similar to that in JAK2, occupying all three sites. However, NSC13626 has different binding conformations both in JAK1 and TYK2. The binding pocket Site 3 of JAK1 and TYK2 are smaller compared to those of JAK2 and JAK3 (Supplementary Figure 9B). The NSC13626 phenol moiety, which occupies Site 3, may be restricted by the narrow pockets observed at Site 3. This further suggests that interactions at Site 3 can increase JAK2 selectivity.

The JAK family have broad functions. For example, JAK1 and JAK2 play roles in hematopoiesis, growth and neural development, while JAK3 and TYK2 play role in the immune responses (O’Shea et al., 2013). Different cytokine receptor subunits are associated with specific JAKs (Banerjee et al., 2017). Aberrant JAK signaling can cause a variety of diseases. For example, increased JAK2 signaling can trigger CRC growth mesenchymal stem cells (Zhang et al., 2018). In contrast, increased JAK1 and JAK3 signaling can lead to T-cell acute lymphocytic leukemia in mice (Degryse et al., 2014). Therefore, a selective blockade of one JAK may inhibit a specific biological function, but allow other JAKs to signal normally. Targeting JAK2, for example, can cause changes to the proliferation, differentiation, survival and apoptosis of myeloid cells (Leroy and Constantinescu, 2017). Blocking of JAK3 may inhibit T cell function. JAK1 and TYK2 have been targeted against autoimmune diseases. Thus, a more specific JAK inhibitor can overcome side effects seen with the pan-JAK blockade.

JAK2 contains two domains, JH1 kinase domain and JH2 pseudokinase domains. A common mutation, V617F, occurs on the JH2 domain and is implicated in MPN. The V617F mutant is located on the JH2 domain and activates the JH1 kinase domain activity (Baxter et al., 2005). Because our studies focused on the JAK2 enzymatic function of JH1 domain, NSC13626 may show efficacy against the JAK2 V617F mutant variant. This mutation occurs on the JH2 domain and can be seen at the cellular level. The effect of NSC13626 against the V617F mutation needs to be further studied.

The binding pose of NSC13626, in JAK2 has similarities to JAK2 inhibitors lestaurtinib, fedratinib, and ruxolitinib. These three kinases were selected to their availability and large-scale testing done in previous studies (Davis et al., 2011). Many of the JAK2 inhibitors target JAK family isoforms or other kinase families, which confirms the difficulties in creating a specific JAK2 inhibitor (Leroy and Constantinescu, 2017). Fedratinib and Lestaurtinib produced roughly similar IC_50_ and *K*_d_ values (Leroy and Constantinescu, 2017). NSC13626 inhibited JAK2 activity 56.6% at a concentration of 10 μM (Supplementary Figure 6). This suggests it has an IC_50_ value approximately 10 μM. Compound, NSC13626, has favorable *K*_d_ binding for both JAK2 and JAK3 (Figure 4). NSC13626 have an IC_50_ value and a *K*_d_ value (6.6 μM) in a similar range. While these results can suggest relative potency of a compound, it does not elucidate for selectivity. For instance, lestaurtinib, fedratinib, and ruxolitinib target a variety of different kinases (Supplementary Figure 10). The inhibitors occupy Site 1 and Site 2 in the binding site (Supplementary Figures 10A–C). In Site 1, each compound contains a different scaffold: lestaurtinib is an indolocarbazole, fedratinib contains an aminopyrimidine scaffold, and ruxolitinib is a pyrrolopyrimidine scaffold. The scaffolds of fedratinib and ruxolitinib form two hydrogen bonds with the oxygen of hinge residue Leu932, while that of lestaurtinib only has a hydrogen-bonding interaction. Compound NSC13626 contains a chloroquinoline scaffold, where the nitrogen on the ring creates a hydrogen bond to the nitrogen of Leu932. Residue Leu932 also forms a hydrogen bond to the heterocyclic rings of the three known JAK2 inhibitors (Supplementary Figures 10A–C). This is due to hydrophobic interactions formed at Site 1. These interactions sandwich the ring in a favorable position to form a hydrogen bond between the nitrogen on the heterocyclic ring and Leu932. In Site 2, NSC13626 contains a diethyl(methyl)amine that forms a hydrogen bond with the carboxyl group of residue Asp994. In contrast, lestaurtinib contains two hydroxy groups to form a hydrogen bond to the oxygen of residue Arg980 (Supplementary Figure 10A). Fedratinib and ruxolitinib contains an erbumine and acetonitrile moiety that occupies Site 2 with hydrophobic interactions (Supplementary Figures 10B,C). However, The JAK inhibitors did not show interactions with Site 3. NSC13626 has a phenol moiety that is able to access the Site 3 pocket. The three JAK2 inhibitors have been known to target a variety of kinases (Leroy and Constantinescu, 2017). For instance, lestaurtinib, an analog to staurosporine, is known to target multiple kinases (Fathi and Levis, 2009). In contrast, NSC13626 was observed to be more specific to JAK2 compared to lestaurtinib, fedratinib and ruxolitinib (Supplementary Figure 10D). This suggests that interactions at Site 3 may increase specificity toward JAK2.

According to the literature, the downstream targets of JAK2 including p-STAT3, p-STAT5, Ras/Raf/MEK/ERK, and PI3K/AKT (Santos and Verstovsek, 2011). However, as our p-Stat3 signaling plays an essential role in CRC (Zhang et al., 2018). Hence, we focused on evaluating the effect of NSC13626 on JAK2/STAT3 pathway in the HCT116 cell line. We also assessed whether NSC13626 has the ability to decrease other JAK2 downstream targets. The phosphorylation of ERK and AKT are representatives of Ras/Raf/MEK/ERK and PI3K/AKT signal pathways, respectively. Figure 7 shows NSC13626 selectively inhibited p-STAT3 expression in HCT116 cell line without affecting p-ERK and p-AKT expressions. We also checked the status of p-STAT5 under NSC13626 treatment; however, p-STAT5 expression was not detected (data not shown). This suggests that the antiproliferative effect of NSC13626 was through JAK2/STAT3 signaling in CRC. Previous research shows that STAT3 inhibitors can disrupt the cell growth in the G0/G1 phase (Turkson et al., 2005). However, it has been reported that attenuating STAT3 signaling can trigger S phase arrest, which is consistent with our results (Timofeeva et al., 2013; Zhu et al., 2014). These differences on the cell cycle may be due to distinct compounds in different cell lines, which may affect various cell cycle-related proteins or checkpoints that tightly modulate cell cycle progression. The effect of NSC13626 on other JAK2 signaling pathways needs to be further investigated.

## Conclusion

In this study, we presented a structure-based virtual screening methodology to identify a novel JAK2 inhibitor. JAK2 is a highly researched enzyme due to its involvement in various diseases. However, a specific JAK2 inhibitor has been difficult to identify due to similarities between the JAK family of enzymes. In this work, we identified six pharmacological interactions using known JAK2 inhibitors. By filtering the NCI compound database based on their docking scores and key residue interactions, we identified a novel compound, NSC13626. Kinase profiling showed compound NSC13626 has high selectivity toward JAK2 and JAK3. Our SAR analysis revealed that compound NSC13626 occupies a hydrophobic pocket within the JAK2 binding site, which resulted in increased activity. In addition, *in vitro* assay in colorectal cells show cell growth inhibition, cell cycle arrest and p-STAT3 reduction when treated with compound NSC13626. This indicates that anticancer activity of NSC13626 stems from its interaction with the JAK2/STAT3 pathway. These results provide an interesting strategy for the identification of a novel JAK2 inhibitor. Increasing inhibitor NSC13626 potency toward JAK2 can be explored in a further study.

## Author Contributions

TL, W-CH, and K-CH conceived and designed the experiments. W-CHF, M-WC, C-DC, Y-YC, H-JT, H-LH, and S-LP prepared the materials and performed the experiments. TL, T-YS, J-HH, and K-CH analyzed the data and results. The manuscript was organized and written by TL, M-WC, and K-CH.

## Conflict of Interest Statement

The authors declare that the research was conducted in the absence of any commercial or financial relationships that could be construed as a potential conflict of interest.

## Footnotes

1 https://software.broadinstitute.org/morpheus/

2 https://www.discoverx.com/services/drug-discovery-development-services/kinase-profiling/kinomescan

3 http://www.thermofisher.com/kinaseprofiling

## Abbreviations

ACD: available chemical directory
CRC: colorectal cancer cell
FBS: fetal bovine serum
JAK: janus kinase
MPN: myeloproliferative neoplasms
NCI: national cancer institute
PAINS: pan-assay interference compounds
PDB: protein data bank
QED: quantitative estimate of drug-likeness
SAR: structure-activity relationship
SD: standard deviation
SRB: sulforhodamine B
TCA: trichloroacetic Acid
