# Supplementary material for: A Novel Selective JAK2 Inhibitor Identified Using Pharmacological Interactions
Source: Front Pharmacol. 2018 Dec 4;9:1379. doi: 10.3389/fphar.2018.01379 (PMC6288363; doi:10.3389/fphar.2018.01379)
Supplement: Supplementary file 1 [file Data_Sheet_1.pdf]

Supporting Information:

A novel selective JAK2 inhibitor identified using pharmacophore interactions

Tony Eight Lin[1,#], Wei-Chun HuangFu[2,3,#], Min-Wu Chao[2], Tzu-Ying Sung[4], Chao-Di Chang[2], Yi-Ying Chen[2], Jui-Hua Hsieh[5], Huang-Ju Tu[6], Han-Li Huang[7], Shiow-Lin Pan[2,3,7] and Kai-Cheng Hsu[2,7*]

[1] Ph.D. Program for Cancer Molecular Biology and Drug Discovery, College of Medical Science and Technology, Taipei Medical University and Academia Sinica, Taipei, Taiwan

[2] Graduate Institute of Cancer Molecular Biology and Drug Discovery, College of Medical Science and Technology, Taipei Medical University, Taipei, Taiwan

[3] Ph.D. Program in Biotechnology Research and Development, Taipei Medical University, Taipei, Taiwan

[4] Institute of Bioinformatics and Systems Biology, National Chiao Tung University, Hsinchu, Taiwan

[5] Kelly Government Solutions, Research Triangle Park, North Carolina, United States of America

[6] School of Pharmacy, College of Medicine, National Taiwan University, Taipei, Taiwan

[7] Biomedical Commercialization Center, Taipei Medical University, Taipei, Taiwan

* Correspondence:
Kai-Cheng Hsu
piki@tmu.edu.tw

# These authors contributed equally to this work.

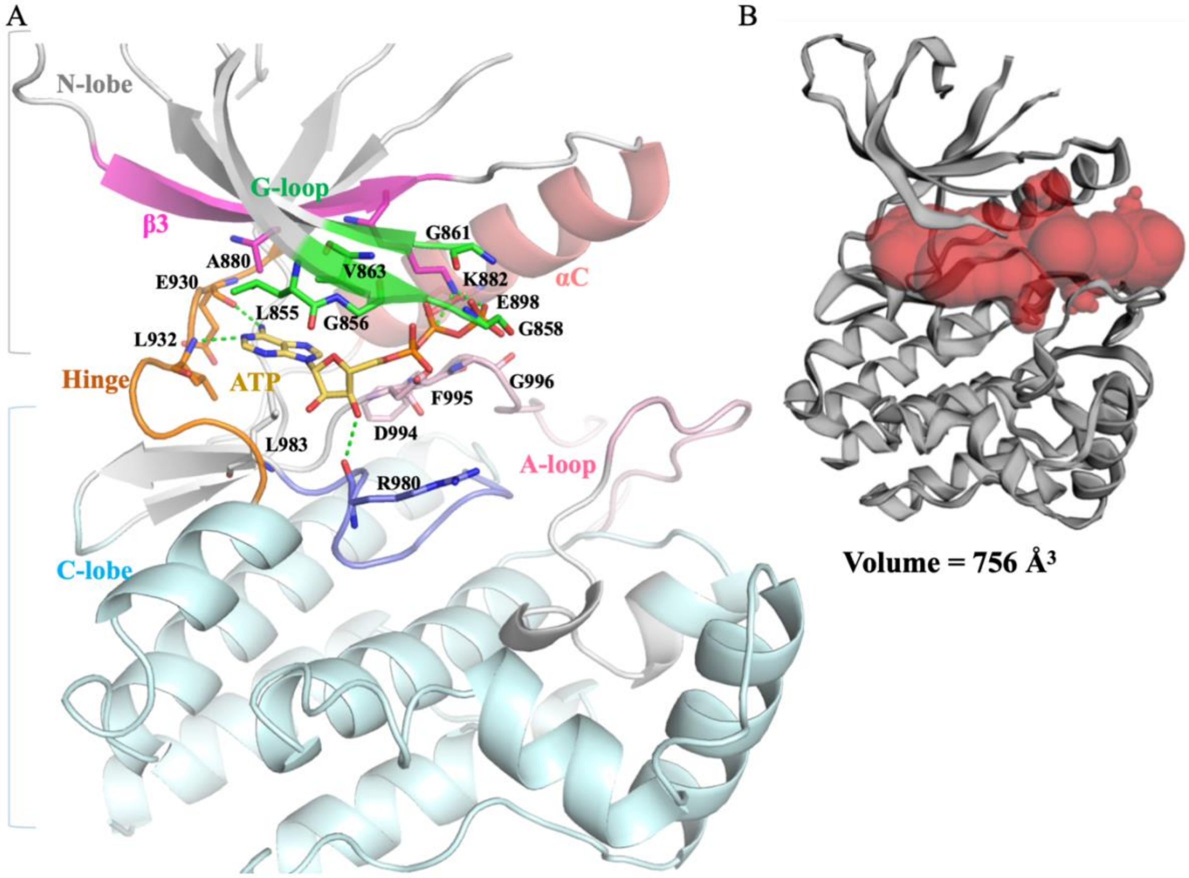

Supplementary Figure 1. JAK2 structure. (A) The JAK2 crystal structure (PDB ID: 3YJ9) rendered in Pymol. The N-lobe and C-lobe are labeled as shown. The Hinge, G-loop, A-loop are colored in orange, green and pink, respectively. (B) The volume (red) of the JAK2 (gray) binding site was calculated using CASTp.

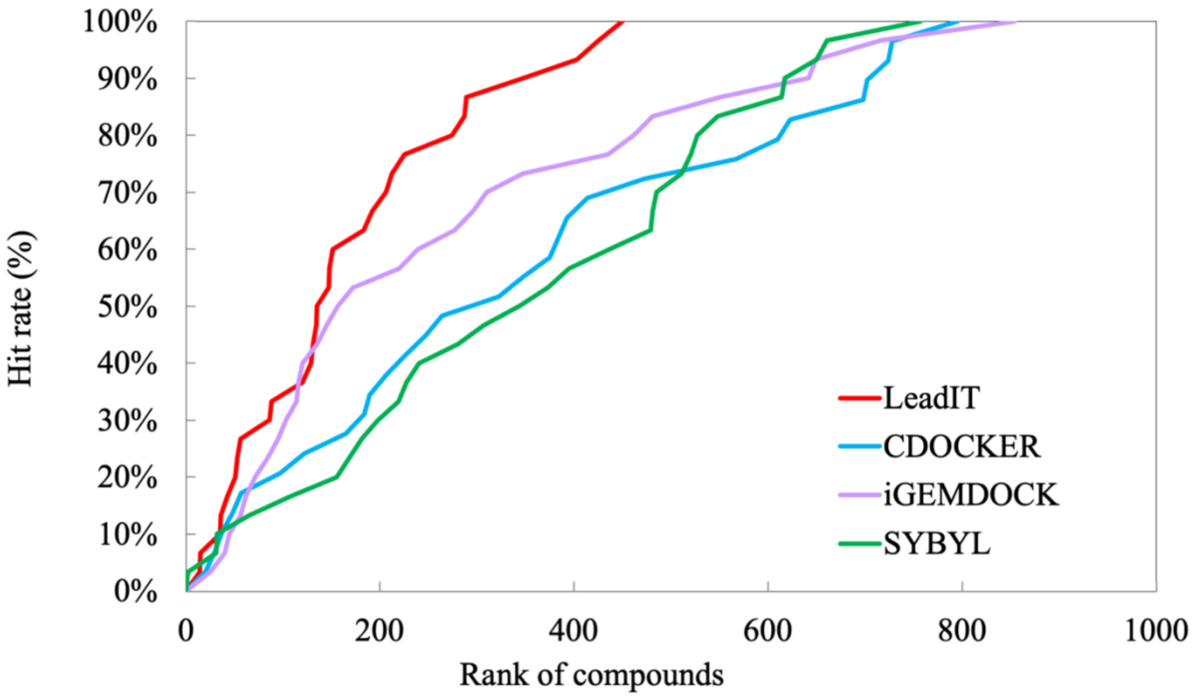

Supplementary Figure 2. Validation of docking software for virtual screening. 30 known JAK2 inhibitors were mixed with 990 ACD compounds, docked in JAK2 using LeadIT, CDOCKER, iGEMDOCK and SYBYL to test for the appropriate docking software for this study.

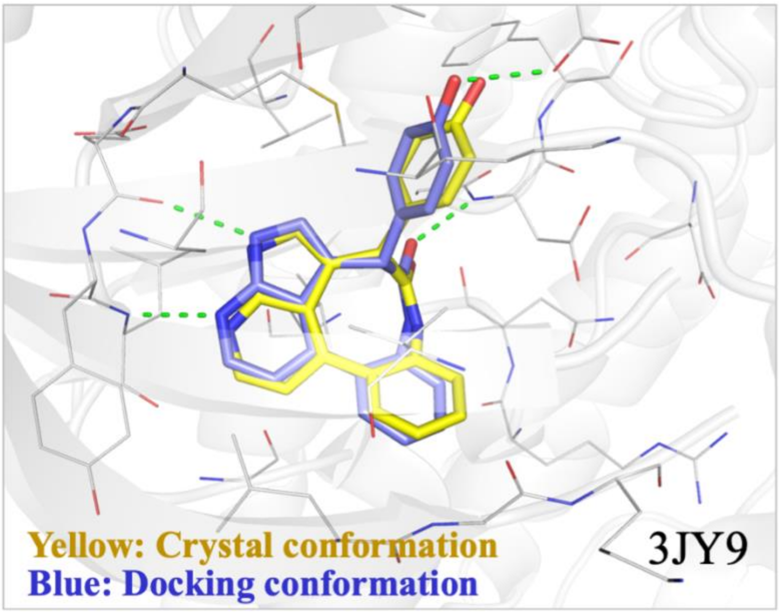

Supplementary Figure 3. Validation of docking procedure by redocking analysis. The co-crystal ligand of JAK2 (PDB ID: 3YJ9) was redocked using LeadIT. The docking pose was superimposed with the co-crystal ligand. No significant spatial difference was observed.

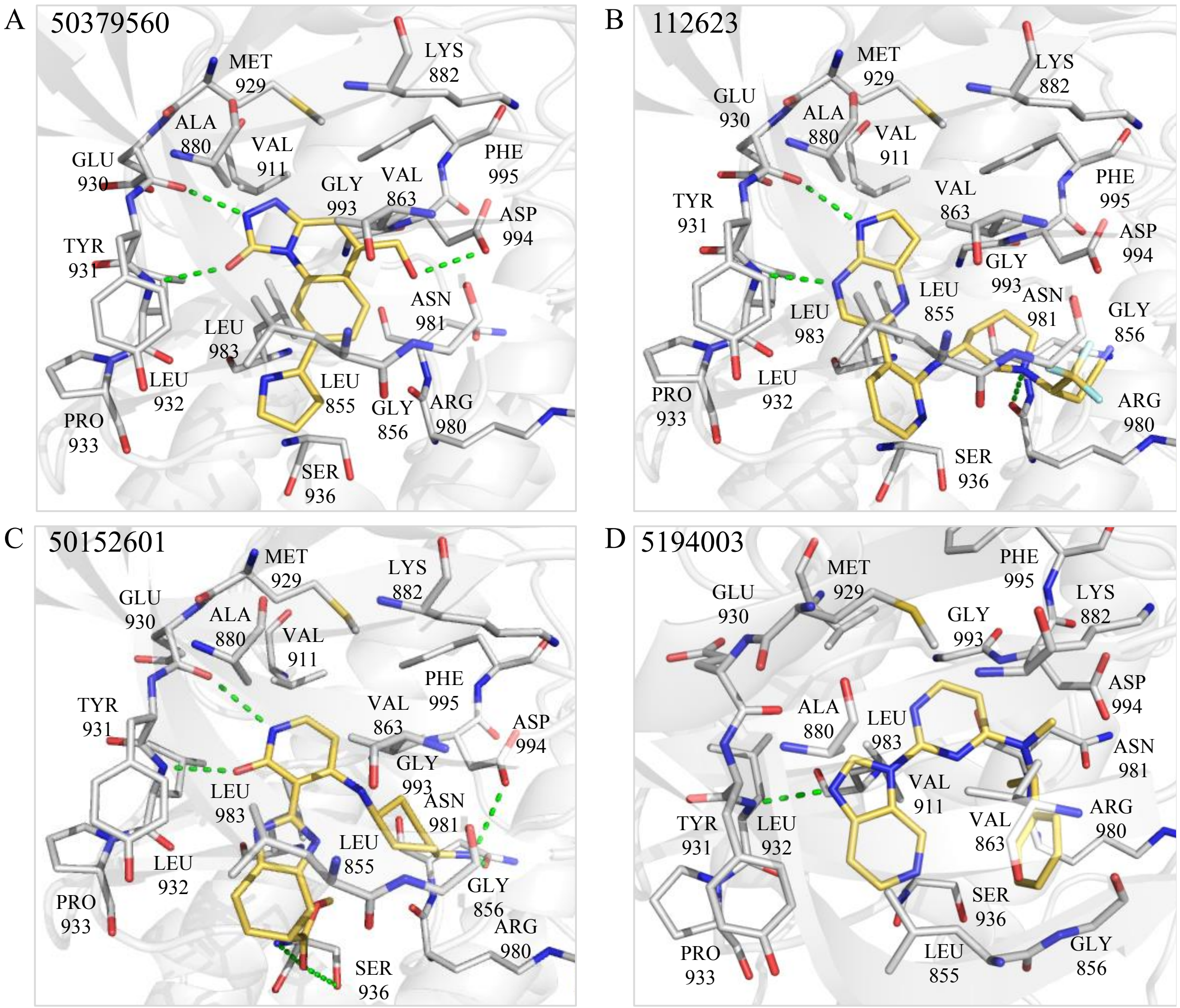

Supplementary Figure 4. Docking poses of known JAK2 inhibitors. Docking poses of known JAK2 inhibitors (yellow) from BindingDB, including 5037960, 112623, 50152601 and 5194003 in JAK2 (gray). Hydrogen bonds are denoted with dash green lines.

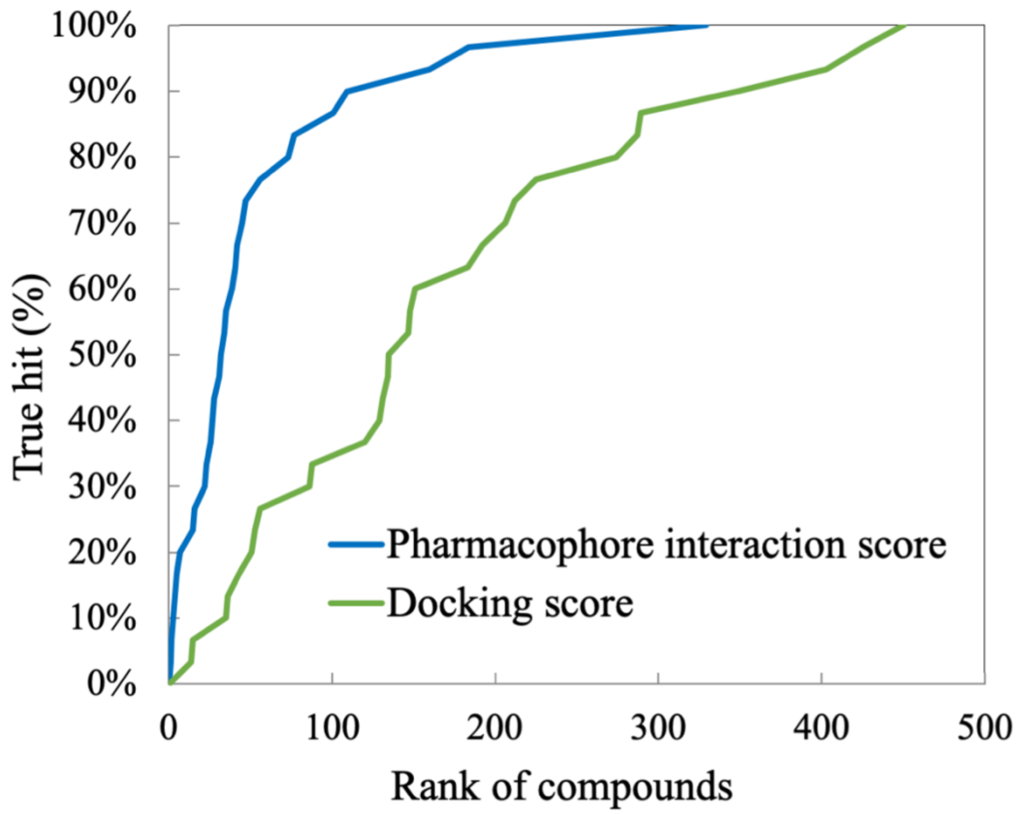

Supplementary Figure 5. Performance comparison of docking score and pharmacological interaction score. 30 known JAK2 inhibitors were mixed with 990 ACD compounds, docked in JAK2 and ranked based on their docking score (green) or number of pharmacological interactions (blue).

Compound	Inhibition percentage at 10 μM	Compound	Inhibition percentage at 10 μM	Compound	Inhibition percentage at 10 μM
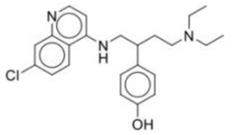 NSC13626	56.5	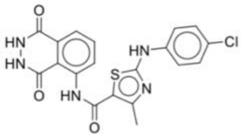 NSC637675	4.6	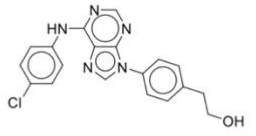 NSC87695	0.6
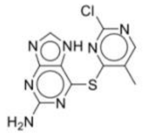 NSC51563	46.1	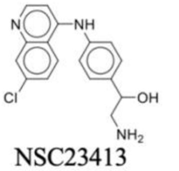 NSC23413	-3.8	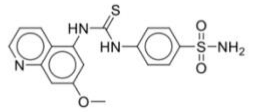 NSC403443	29.4
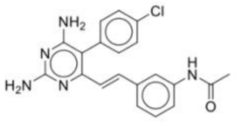 NSC211653	0.5	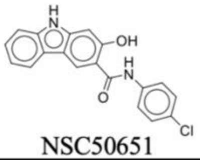 NSC50651	2.3	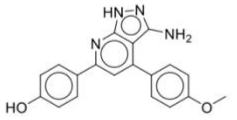 NSC734136	4.0

Supplementary Figure 6. Structures and inhibitory percentages of potential inhibitors. The methods yielded 9 compounds with potential JAK2 inhibitions. Their inhibition percentages and structures are listed as shown.

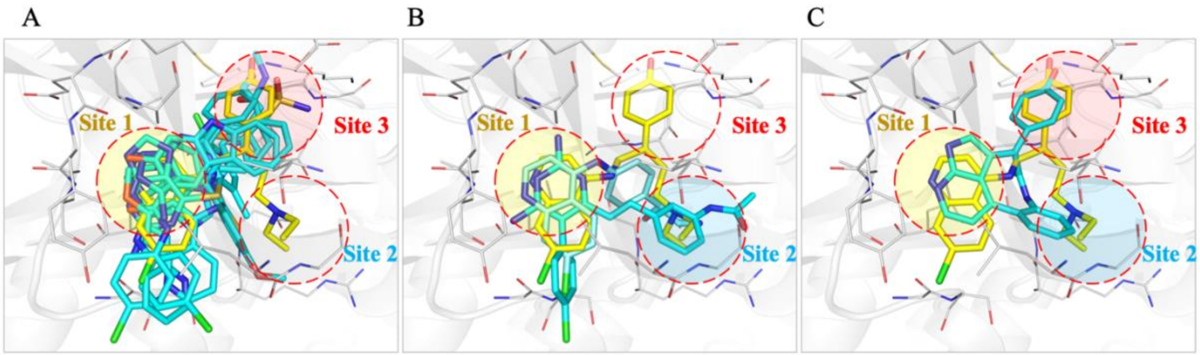

Supplementary Figure 7. Comparison between inactive compounds and NSC13626. (A-B) The docking poses of the virtual hit compounds (blue) superimposed with the active compound NSC13626 (yellow) in JAK2 (gray). (A) The first group contains compounds, NSC51563, NSC87695, NSC734136, NSC50651, NSC637675 and NSC403443, and (B) the second group contains compounds NSC211653 and NSC23413. (C) The binding conformation of the co-crystal ligand, JZH (blue) superimposed with NSC13626.

Compound	Inhibition percentage at 10 μM	Compound	Inhibition percentage at 10 μM
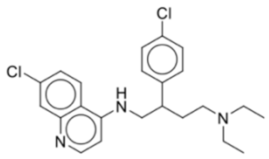<br>NSC10036	2	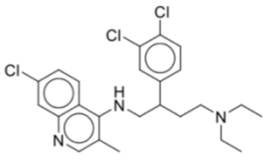<br>NSC13630	-9
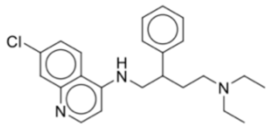<br>NSC10037	1	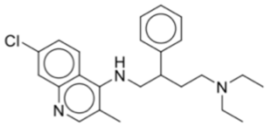<br>NSC11052	3
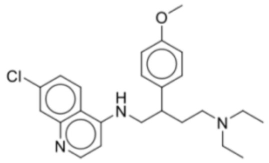<br>NSC10493	1	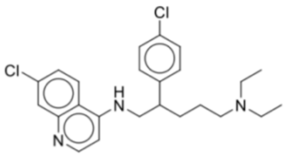<br>NSC10492	-2
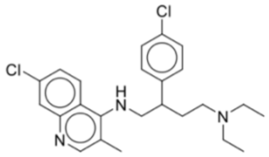<br>NSC13724	-1	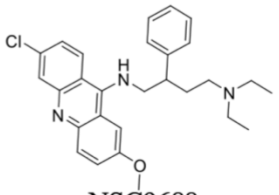<br>NSC9688	3

Supplementary Figure 8. Structures and inhibitory percentages of NSC13626 analogs. Analogs to NSC13626 in the NCI database were found. All analogs showed weak JAK2 inhibition at 10 μM.

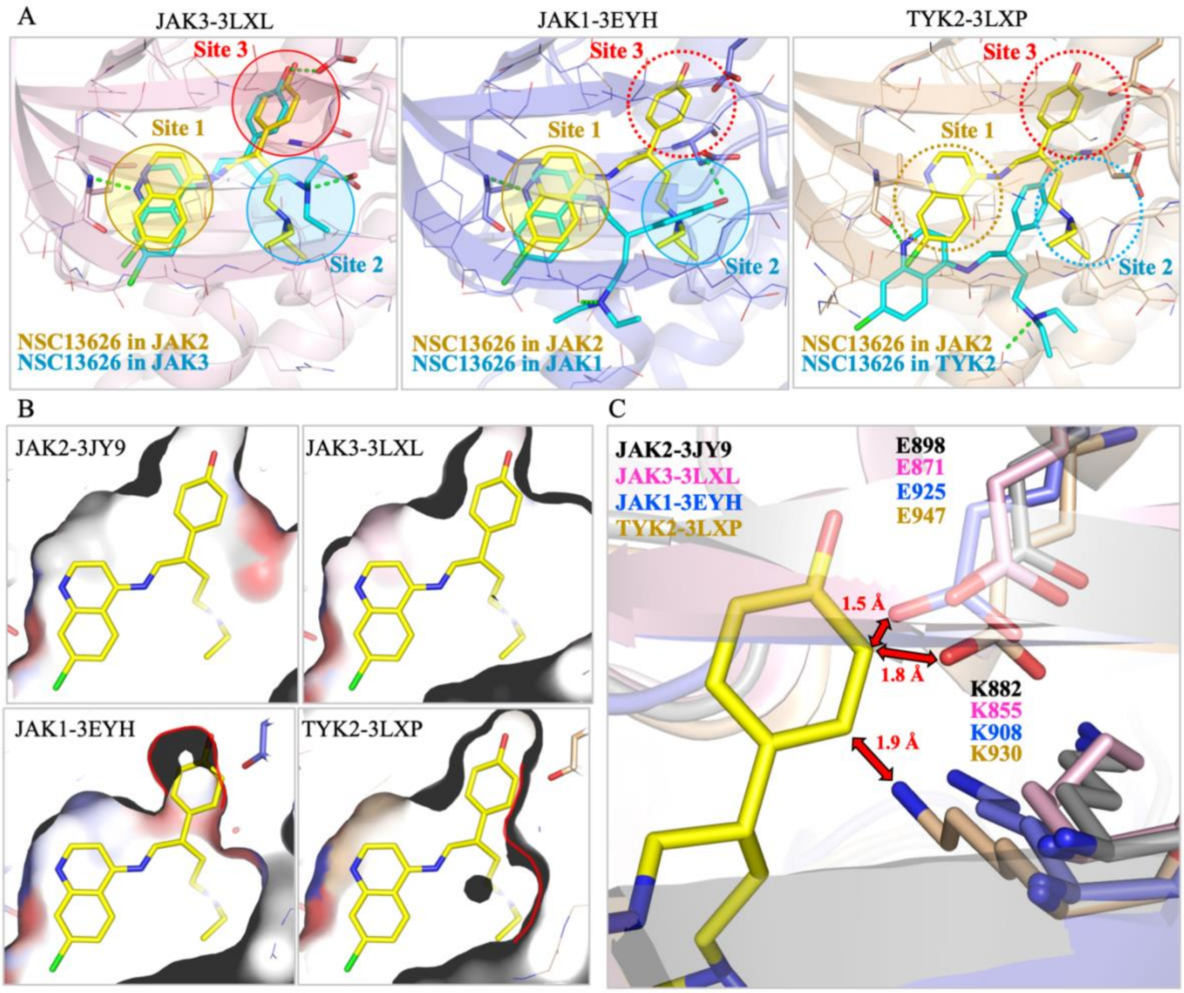

Supplementary Figure 9. The docking pose of NSC13626 in JAK family. (A) The docking pose of NSC13626 in JAK3 (pink), JAK1 (blue) and TYK2 (yellow) with the docking pose of NSC13626 superimposed. No significant differences were observed between the docking pose of JAK2 and JAK3. In contrast, JAK1 and TYK2 showed significant differences. (B) The binding pocket of the JAK family with the docking pose of NSC13626 from JAK2. A significant reduction with the binding site is observed with JAK1 and TYK2. (C) The distance between glutamate and lysine residue and the phenol moiety in JAK1 and TYK2 does not produce effective interactions.

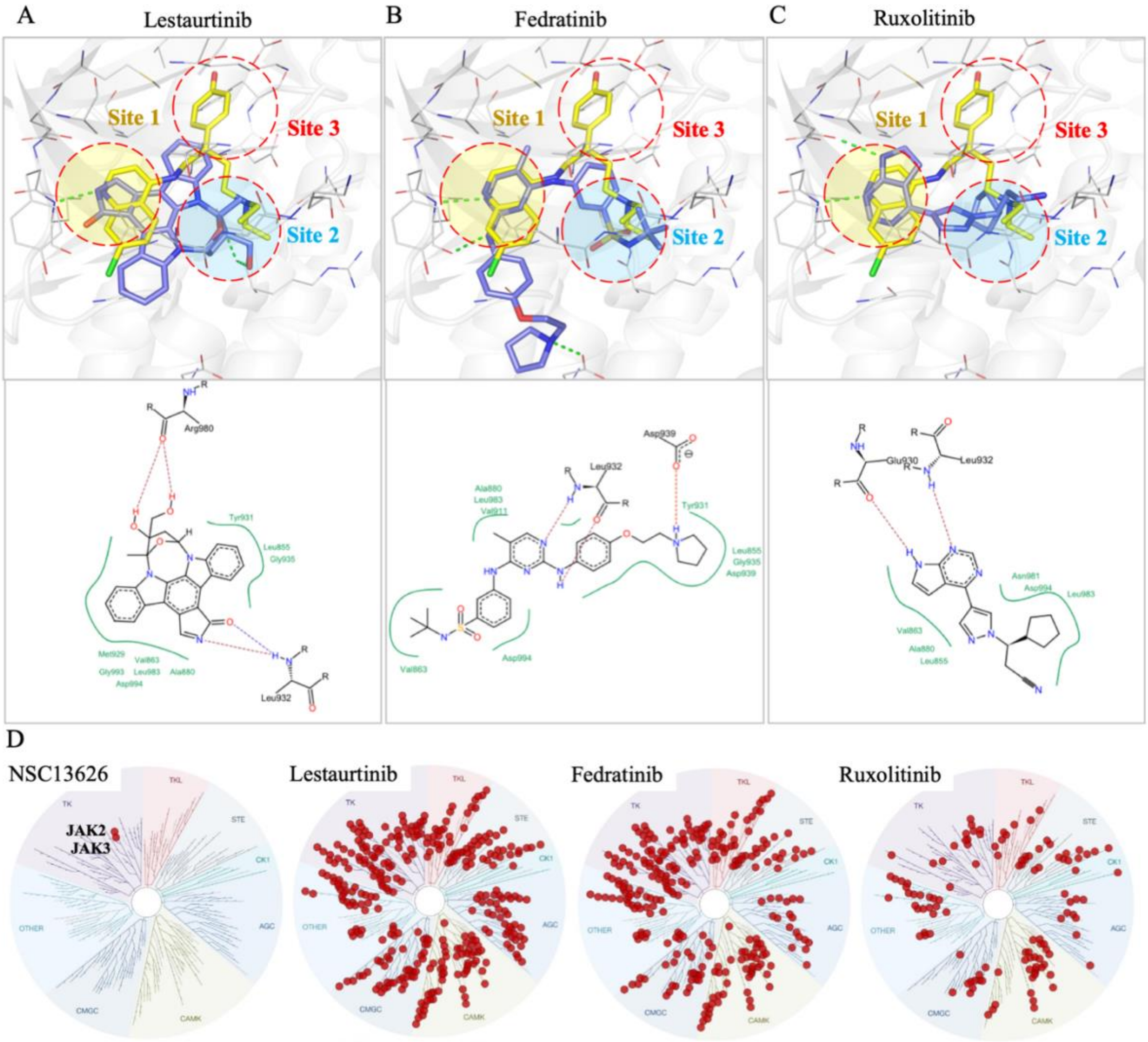

Supplementary Figure 10. Docking pose comparison between known JAK2 inhibitors and NSC13626. The docking pose of NSC13626 (yellow) in JAK2 is compared between lestaurtinib (A), fedratinib (B) and ruxolitinib (C). Their 3D poses were generated in Pymol, while their 2D representations were generated in LeadIT. (D) NSC13626 showed selectivity towards JAK2 and JAK3, while lestaurtinib, fedratinib and ruxolitinib can target a variety of kinases. Red circle indicates kinase targets for the compounds with K_d values of $\leqq 10\ \mu M$. The figures were generated using TREEspot (http://treespot.discoverx.com/)
